# The Oncometabolite 5′-Deoxy-5′-Methylthioadenosine Blocks Multiple Signaling Pathways of NK Cell Activation

**DOI:** 10.3389/fimmu.2020.02128

**Published:** 2020-10-06

**Authors:** Benedikt Jacobs, Sebastian Schlögl, Carolin Dorothea Strobl, Simon Völkl, Andrej Stoll, Dimitrios Mougiakakos, Karl-Johan Malmberg, Andreas Mackensen, Michael Aigner

**Affiliations:** ^1^Department of Internal Medicine 5, Hematology and Oncology, Friedrich Alexander University Erlangen-Nuremberg (FAU), University Hospital Erlangen, Erlangen, Germany; ^2^Department of Anesthesiology, Intensive Care and Pain Therapy, General Hospital Fürth, Fürth, Germany; ^3^Center for Infectious Medicine, Department of Medicine, Karolinska Institutet, Stockholm, Sweden; ^4^K.G. Jebsen Center for Cancer Immunotherapy, Institute of Clinical Medicine, University of Oslo, Oslo, Norway; ^5^Department of Cancer Immunology, Institute for Cancer Research, Oslo University Hospital, Oslo, Norway

**Keywords:** NKG2C, CD16 signaling, 5′-deoxy-5′-methylthioadenosine, NK cells, tumor escape mechanism

## Abstract

Tumor cells develop various mechanisms to escape immune surveillance. In this context, oncometabolites secreted by tumor cells due to deregulated metabolic pathways, have been in the spotlight of researchers during the last years. 5′-Deoxy-5′-methylthioadenosine (MTA) phosphorylase (MTAP) deficiency in tumors results in the accumulation of MTA within the tumor microenvironment and thereby negatively influencing immune functions of various immune cells, including T and NK cells. The influence of MTA on T cell activation has been recently described in more detail, while its impact on NK cells is still largely unknown. Therefore, we aimed to illuminate the molecular mechanism of MTA-induced NK cell dysfunction. NK cell cytotoxicity against target cells was reduced in the presence of MTA in a dose-dependent manner, while NK cell viability remained unaffected. Furthermore, we revealed that MTA blocks NK cell degranulation and cytokine production upon target cell engagement as well as upon antibody stimulation. Interestingly, the immune-suppressive effect of MTA was less pronounced in healthy donors harboring an expansion of NKG2C^+^ NK cells. Finally, we demonstrated that MTA interferes with various signaling pathways downstream of the CD16 receptor upon NK cell activation, including the PI3K/AKT/S6, MAPK/ERK, and NF-κB pathways. In summary, we revealed that MTA blocks NK cell functions like cytotoxicity and cytokine production by interfering with the signaling cascade of activating NK cell receptors. Specific targeting of MTA metabolism in MTAP-deficient tumors therefore could offer a promising new strategy to reverse immune dysfunction of NK cells within the tumor microenvironment.

## Introduction

Altered tumor cell metabolism is able to contribute to various tumor escape mechanisms to evade destruction by the immune system. An elevated glycolysis rate leads to increased lactic acid production, resulting in its accumulation within the tumor microenvironment (TME), where it blocks IFNγ production and reduces survival of CD8^+^ T and NK cells *in vitro* and *in vivo* (1). Moreover, tumor cells often favor metabolic pathways leading to the accumulation of intermediate metabolites; one example for these so-called oncometabolites is 2-hydroxyglutarate (2-HG), which is released by tumor cells harboring a gain-of-function mutation of the isocitrate dehydrogenase (IDH). Accumulation of 2-HG results in increased uptake by T cells, reprogramming their metabolism toward oxidative phosphorylation, leading to an increased frequency of regulatory T cells (Treg) and impaired polarization of T helper 17 (Th17) cells (2). Furthermore, various tumor entities have shown a reduced activity of the 5′-deoxy-5′-methylthioadenosine phosphorylase (MTAP), an important enzyme of the polyamine and methionine salvage pathway, either due to promoter hypermethylation or deletion of the chromosomal 9p21 region (3–5). MTAP is the only human enzyme that converts 5′-deoxy-5′-methylthioadenosine (MTA), a by-product of the polyamine pathway, into adenine and 5′-methylthioribose-1-phosphate. The latter one is then further metabolized to methionine within the methionine salvage pathway, which assures a sufficient production of S-adenosyl-methionine (SAM/AdoMet), the most important methyl donor within eukaryotic cells. Proper removal of MTA by MTAP is essential to guarantee an effective performance of the polyamine synthesis pathway and of methylation processes (6). We have previously demonstrated that accumulation of MTA due to MTAP deficiency is able to suppress proliferation, activation, and differentiation of human T cells (7, 8). In addition, an immune-suppressive effect of MTA has been demonstrated as well within cells of the innate immune system including macrophages (9, 10) and NK cells (11).

NK cells are innate lymphocytes, which, in contrast to T and B cells, recognize their targets through a variety of germline-encoded activating and inhibitory receptors. In this regard, tumor or virus-infected cells often down-regulate human leucocyte antigen (HLA) molecules on their surface in order to escape the adaptive immune system. However, HLA molecules like HLA-C1, C2, Bw4, or E are all ligands for inhibitory NK cell receptors like killer immunoglobulin-like receptor (KIR; HLA-C1, C2, Bw4) or NKG2A (HLA-E). Thus, down-regulation of HLA molecules with resulting predominance of activating receptors on target cells renders these cells susceptible toward NK cell cytotoxicity, a mechanism called “missing-self” (12). In addition, NK cells produce proinflammatory cytokines like interferon gamma (IFNγ) and tumor necrosis factor alpha (TNF) upon encountering a target cell, thereby inducing direct as well as indirect anti-tumor effects like the activation and differentiation of naïve T cells (13). NK cells are characterized by the lack of a TCR and its CD3 co-receptor while expressing the FcγRIII receptor CD16 and CD56; density and expression are both used for the additional division into the immature CD56^bright^CD16^+/−^ and the mature CD56^dim^CD16^+^ NK cell subsets (14). The latter one can be further divided based on the expression of NKG2A, KIR, and CD57 (15). Recently, a NK cell subset with adaptive immune features has been described in CMV-infected individuals. These cells demonstrate longevity, clonal expansion, and enhanced effector function and were transplantable into other individuals. They exhibited increased expression of the activation receptor NKG2C and of the terminal differentiation marker CD57 (16–18).

The current project aimed to explore the underlying mechanism of how MTA is blocking NK cell cytotoxicity in order to further understand this process in detail and develop new strategies to circumvent this tumor escape mechanism.

## Materials and Methods

### Reagents and Cell Lines

Antibodies were purchased for CD16 biotin from BioLegend; LFA-1 open conformation isoform was from Abcam; pZAP/Syk, pS6, pSLP76, pAKT (S473), pPLCγ2, pERK1/2, and NF-κB pp65 were from BD; KIR2DL1/S1 was from Miltenyi; KIR2DL2/3/S2 was from Beckman Coulter; KIR3DL1/2 was from BioLegend; CD57 was from BioLegend; NKG2A was from Beckman Coulter; NKG2C was from Miltenyi; CD56 was from Beckman Coulter; CD16 was from BioLegend; 7AAD was from BD; dead-cell marker was from Life Technologies; and CD107a was from BioLegend. Pacific Orange and Blue Succinimidyl Ester were bought from Thermo Fisher Scientific. 5-Methylthioadenosine (MTA) and 3-deazaadenosine (3-Deaza) were purchased from Sigma-Aldrich, 5-azacytidine (5-Aza) was from Biomol/Cayman, and 2-chloroadenosine (CADO) and EPZ015666 (EZH) were from Sigma. Avidin was purchased from Sigma. K562 cell line from ATCC was cultured in RPMI 1640 media with antibiotics (penicillin/streptomycin; Invitrogen) and 10% heat-inactivated fetal calf serum (Sigma) at 37°C.

### Blood Donors and PBMC Isolation

Blood from healthy volunteer donors were obtained from the Erlangen and Oslo University Hospital Blood Bank with written donor informed consent. Peripheral blood mononuclear cells (PBMCs) were isolated using density gravity centrifugation (Lymphoprep; Axis-Shield). Isolated PBMCs were frozen down in freezing media [90% fetal calf serum and 10% DMSO] at −80°C and transferred into a liquid nitrogen tank for long-term storage.

### NK Cell Isolation and Culture

Frozen PBMCs were thawed and washed before they were used for NK cell isolation. NK cell isolation from fresh or frozen PBMCs was performed using a NK cell isolation kit and magnetic column separation technology (Miltenyi Biotec). Isolated NK cells were either directly used for functional assays (phospho-epitope analysis) or rested overnight in complete medium containing RPMI 1640 media with antibiotics (penicillin/streptomycin; Invitrogen) and 10% heat-inactivated fetal calf serum (Sigma) plus 100 U/ml IL-2 (Proleukin) at 37°C.

### ^51^Cr Cytotoxic Assay

A total of 1 × 10^6^ K562 cells were incubated with 100 μCi ^51^Cr for 2 h at 37°C. Afterwards, K562 cells were intensively washed and co-incubated at a 5:1 ratio with NK cell, which has been rested overnight with 100 U/ml IL-2 and pre-incubated with different concentrations of MTA. Cells were incubated for 4 h in the presence of increasing MTA concentrations at 37°C. At the end of the culture, cells were pelleted and 100 μl of supernatant was taken away for measuring radioactivity at a Liquid Scintillation counter (Perkin Elmer). NK cell cytotoxicity was calculated as % specific lysis = [(sample cpm–spontaneous cpm)/(maximal cpm–spontaneous cpm)]^*^100 (19). Maximum release was achieved by incubating K562 cells in perchloric acid and spontaneous release by incubation in complete medium alone.

### Annexin V Staining Assay

NK cells rested overnight with 100 U/ml IL-2 were incubated with increasing concentrations of MTA in complete medium without additional IL-2 for 4 h at 37°C. Cells were harvested, washed, and labeled in 100 μl of 1 × Annexin V Binding Buffer (BD Biosciences) with 5 μl of Annexin V-FITC and 7AAD at room temperature for 15′. Afterwards, 400 μl of 1 × Annexin V buffer was added and cells were analyzed at a FACSCanto II machine (BD Biosciences). FlowJo software was used for analyzing FACS data (FlowJo LLC).

### Flow Cytometry-Based Functional NK Cell Assays

Isolated NK cells from thawed PBMCs were rested overnight with 100 U/ml IL-2 at 37°C and pre-incubated with various concentrations of MTA or other inhibitors for 30′ at 37°C. Afterwards, NK cells were stimulated for 4 h with either plate-bound CD16 antibodies (10 μg/ml CD16-biotin antibodies were attached to an Avidin-coated 96-flat bottom plate for 15′ at room temperature) or K562 cells at a 1:1 ratio at 37°C in complete medium. CD107a was directly pipetted into the culture medium. After 1 h, GolgiPlug™/ Stop™ (BD) were added to the culture. After 4 h, cells were harvested and stained for surface epitopes. Afterwards, cells were fixed, permeabilized, and stained for intracellular cytokines using the fixation/permeabilization solution kit (BD).

### Conjugate Formation Assay

Overnight rested NK cells were labeled with the VPD450 dye (BD) for 10′. K562 cells were labeled with the PKH26 dye according to the manufacturer's instruction (Sigma-Aldrich). VPD450-labeled NK cells were pre-incubated with or without 100 μM MTA for 30′ at 37°C. Afterwards, VPD450-labeled NK cells and PKH26-labeled K562 cells were put together into a FACS tube and spin down for 1′ at 100*g*. Cells were then incubated in a water bath at 37°C for 10′ and directly fixed in 300 μl of paraformaldehyde on ice. Finally, cells were washed and analyzed at a FACSCanto II machine (BD Biosciences).

### Inside-Out Signaling Assay

Isolated NK cells from thawed PBMCs were rested overnight with 100 U/ml IL-2 at 37°C and pre-incubated with or without 100 μM MTA for 30′ at 37°C. NK cells were then stimulated with K562 cells for 10′ at 37°C at a 1:1 ratio. Afterwards, cells were harvested and stained for NK cell surface markers and the LFA-1 open conformation isoform.

### Fluorescent Cell Barcoding and Phospho-Epitope Staining

NK cells isolated from fresh PBMCs were pre-incubated with or without 100 μM MTA in complete medium at 37°C for 30′. NK cells were then labeled with 10 μg/ml CD16-biotin antibodies for 2′ in a water bath at 37°C. Afterwards, 50 μg/ml avidin was added to cross-link the antibodies and induce the CD16 signaling cascade. After 0, 1, 5, 10, and 30′, NK cells were fixed in 4% paraformaldehyde for 7′ at 37°C, pelleted, washed, and stored in 100% methanol at −20°C. After overnight storage, cells were thawed, washed, and labeled with different concentrations of different dilution combinations of the two fluorescent dyes pacific blue and orange (Life Technologies). Afterwards, all cells were collected, labeled with surface and different phospho-epitope specific antibodies, and analyzed at a FACSCanto II machine (BD Biosciences).

### Microarray Analysis

The dataset GSE23695 containing expression profiles of CD56^dim^CD16^+^CD57^+^ and CD57^−^ NK cells was downloaded into R version 3.6.1 using the package GEOquery 2.54.1 (20). Subsequent differential expression analysis was carried out with limma 3.42.2. Gene set enrichment analysis (GSEA) was performed with GSEA v4.0.3 on gene set M10911 (KEGG_CYSTEINE_AND_METHIONINE_METABOLISM).

### Statistical Analysis

For comparing single paired samples, a Wilcoxon test was used, while a Mann–Whitney test was used for single unpaired samples. For significance analysis for multiple matched groups with each other, a one- or two-way ANOVA test was done. Statistical significance: ^*^p < 0.05. Analysis was performed using the GraphPad Prism software.

## Results

### MTA Blocks NK Cells' Cytotoxic Activity Without Affecting Their Viability

**Figure 1:**
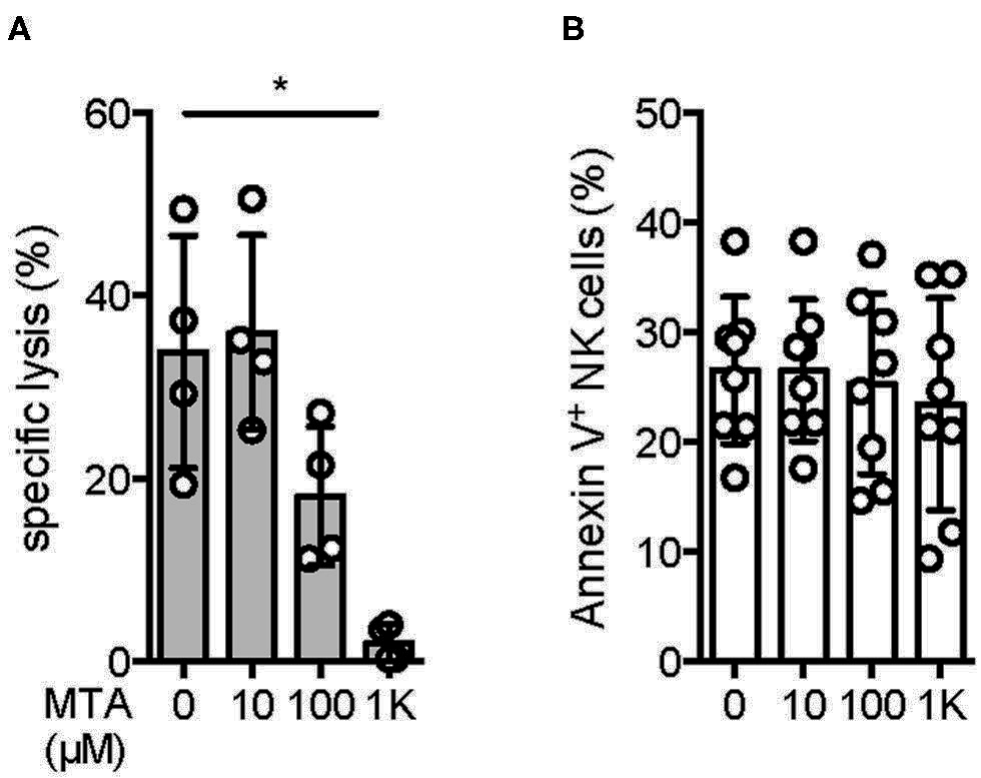
NK cells' cytotoxic activity is reduced upon MTA co-incubation without affecting NK cell viability. Isolated NK cells were incubated overnight with 100 U/ml IL-2 and pre-incubated for 30′ with various concentrations of MTA at 37°C. NK cells were further incubated for 4 h with the prior MTA concentration in the presence **(A)** or absence **(B)** of ^51^Cr-labeled K562 cells at a 5:1 ratio. For evaluating NK cells' cytotoxic activity against K562 cells (**A**; *n* = 4), the % specific lysis was calculated as followed: [(sample cpm–spontaneous cpm)/(maximal cpm–spontaneous cpm)]^*^100. To analyze the effect of MTA on NK cells' viability, they were harvested and stained for Annexin V expression (**B**; *n* = 8). Significance was calculated using a one-way ANOVA test (*p* value: ^*^ <0.05).

Since NK cells are known for their cytotoxic activity against various tumor cell lines, we were interested if MTA is capable of suppressing this effect. Using the well-established NK cell target cell line K562 in a classical ^51^Cr release assay, we observed a reduction of NK cell cytotoxic activity at a MTA concentration of 100 μM, with no suppressive effect at 10 μM. In contrast, a nearly complete block of cytotoxicity was found at a concentration of 1 mM MTA (Figure 1A). To rule out a cytotoxic effect of MTA on NK cells' viability as a reason for this reduced cytotoxic activity, we stained NK cells for Annexin V and 7AAD expression after a 4 h incubation period with increasing concentrations of MTA. Interestingly and importantly, even at a MTA concentration of 1 mM, no increased Annexin V (Figure 1B) or 7AAD (Supplementary Figure 1A) expression was observed. Overall, NK cell cytotoxic activity against K562 cells is reduced with increasing MTA concentrations which is not due to a cytotoxic MTA effect on NK cells' viability however.

### NK Cell Degranulation and Cytokine Production Are Reduced by MTA

**Figure 2:**
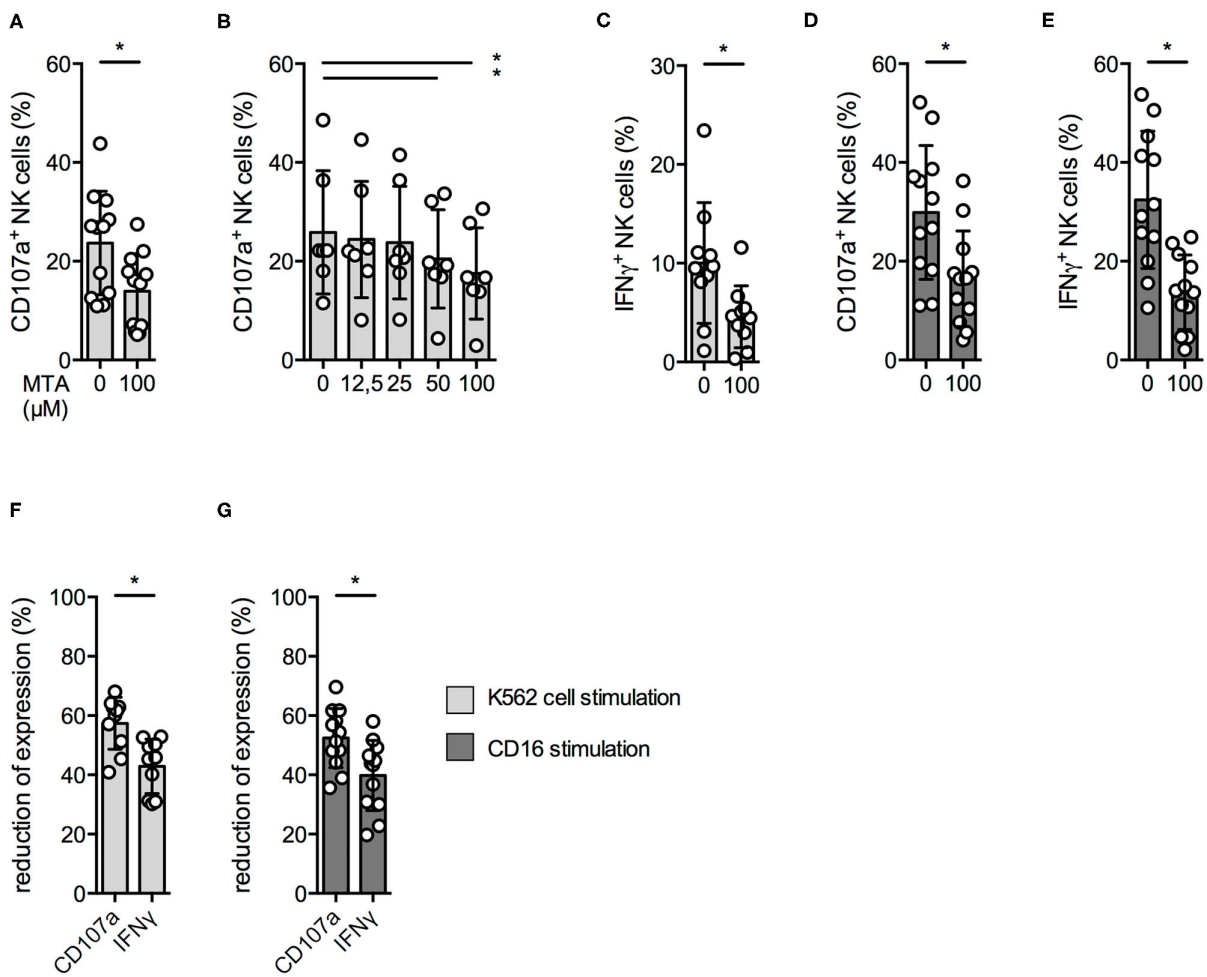
NK cell degranulation and cytokine production are reduced by MTA. Overnight IL-2-activated NK cells were pre-incubated with different concentrations of MTA for 30′ at 37°C and then stimulated with K562 cells at a 1:1 ratio (**A–C,F**) or plate-bound CD16 (**D,E,G**) for additional 4 h at 37°C. Afterwards, the cells were harvested and CD107a or IFNγ expression was analyzed in bulk NK cells. CD107a and IFNγ expression were indicated either as absolute values **(A–E)** or as the percentage decrease **(F,G)** due to MTA co-incubation [(absolute value with MTA/absolute value without MTA)*100]. Significance was calculated using a Wilcoxon test for analyzing paired samples (**A,C–G**; *n* = 12) or a one-way ANOVA (**B**; *n* = 6) test (*p* value: * <0.05).

Upon tumor cell recognition, NK cells release the content of their cytotoxic granules by a process called degranulation and produce pro-inflammatory cytokines like IFNγ. Since we observed a reduced cytotoxic activity at 100 μM MTA, we investigated if this was due to a reduced NK cell degranulation upon contact with K562 cells. Indeed, CD107a surface expression—a degranulation marker—on NK cells was significantly reduced in the presence of 100 μM MTA (Figure 2A); this could be observed even at lower MTA concentrations (Figure 2B). In accordance to our cytotoxicity experiments, NK cell degranulation in the presence of target cells was almost completely blocked at 1,000 μM MTA (Supplementary Figure 2A) and, in addition, IFNγ production was significantly reduced as well (Figure 2C).

As NK cells can also be activated by binding of antibodies to their CD16 receptor, we also investigated this stimulatory pathway. MTA was able to significantly reduce CD107a expression and IFNγ production upon CD16 stimulation (Figures 2D,E), while CD16 surface expression remained unchanged (Supplementary Figure 2B).

Of note, IFNγ production was more impaired by MTA than degranulation, independently if stimulated by either K562 cells (Figure 2F) or anti-CD16 (Figure 2G). Altogether, MTA-induced suppression of NK cells' cytotoxic activity is due to a reduced NK cell degranulation; in addition, MTA blocks IFNγ production upon various stimuli.

### Adenosine Derivates and PRMT5 Inhibitor Reduce NK Cell Function

In other immune cell populations, it has been shown that MTA exerts its suppressive effect by different mechanisms. One supposed mechanism is the interaction with adenosine receptors due to the adenosine residue in MTA and another one due to its intracellular inhibition of the protein arginine methyltransferase 5 (PRMT5) (8, 21, 22).

**Figure 3:**
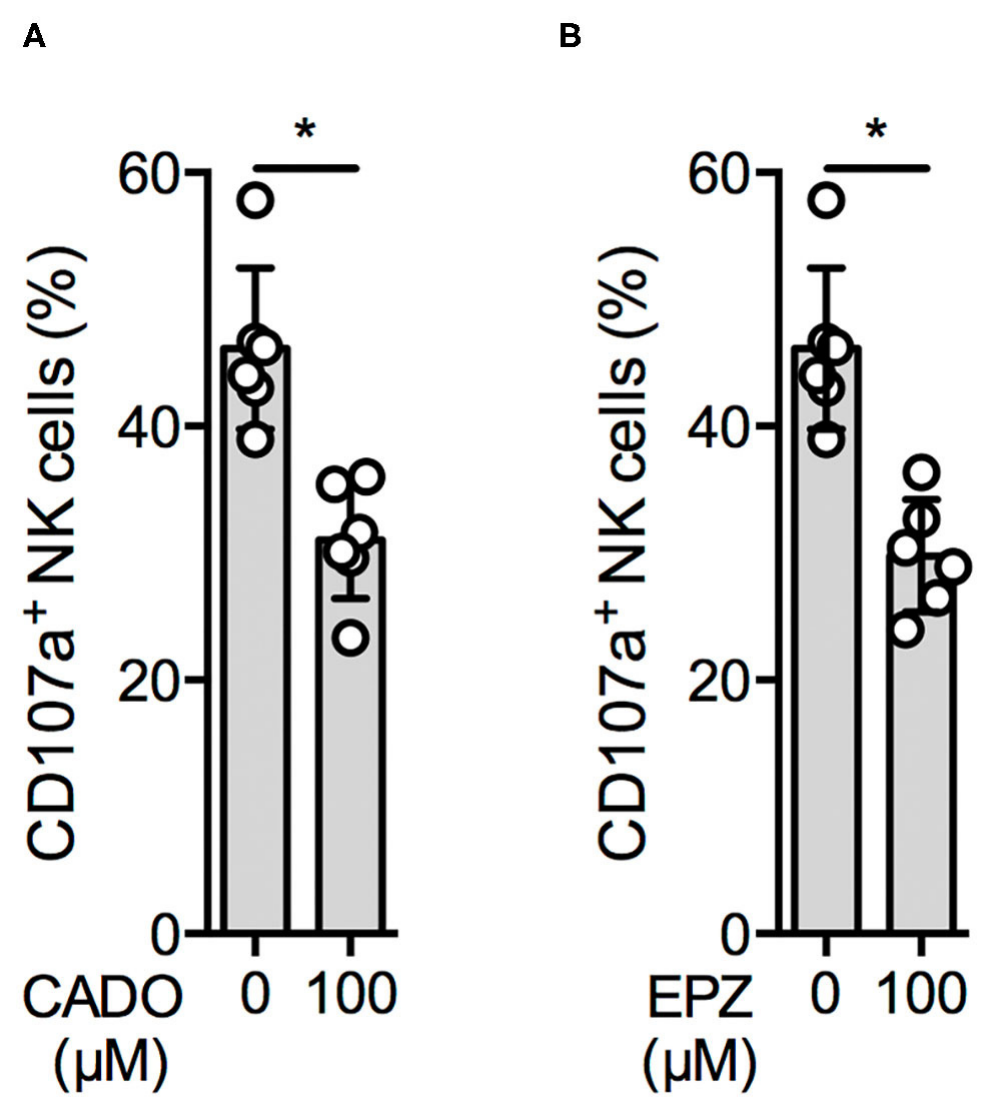
An adenosine derivate and a PRMT5 inhibitor reduce NK cell function. Overnight IL-2-activated NK cells were pre-incubated for 30′ with 100 μM 2-chloroadenosine (CADO) or EPZ015666 (EPZ) (**A,B**; *n* = 6) and then stimulated with K562 cells at a 1:1 ratio for additional 4 h at 37°C. Afterwards, the cells were harvested and CD107a expression was analyzed in bulk NK cells. CD107a expression was indicated as absolute values **(A,B)**. Significance was calculated using a Wilcoxon test for analyzing paired samples (*p* value: * <0.05).

We therefore investigated NK cell degranulation upon K562 cell stimulation in the presence of the stable adenosine analog 2-chloroadenosine (CADO) (23) and a synthetic PRMT5 inhibitor, EPZ015666 (EPZ). CD107a surface expression was significantly reduced in the presence of 100 μM CADO (Figure 3A) and 100 μM EPZ (Figure 3B). A similar effect was observed using the protein methyltransferase inhibitor 3-deazaadenosine (Supplementary Figure 3A), whereas no significant reduction was observed using the DNA methyltransferase inhibitor 5-azacitidine (Supplementary Figure 3B).

### MTA Elicits Different Inhibitory Effects Within the Different NK Cell Subsets

**Figure 4:**
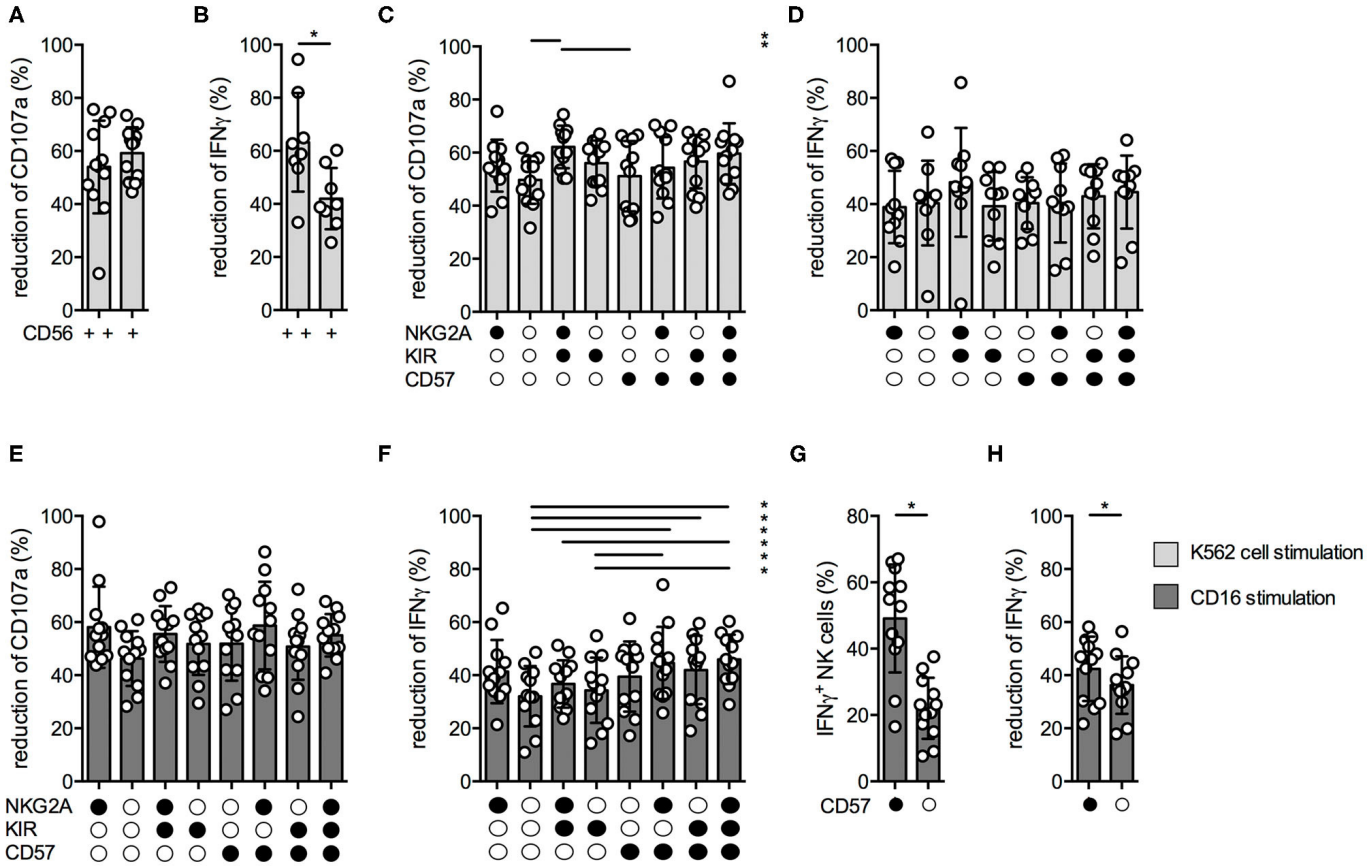
MTA's inhibitory effect varies within the different NK cell subsets. Overnight IL-2-activated NK cells were pre-incubated with 100 μM MTA for 30′ at 37°C and then stimulated with K562 cells at a 1:1 ratio **(A–D)** or plate-bound CD16 **(E–H)** for additional 4 h at 37°C. Afterwards, the cells were harvested and CD107a or IFNγ expression were analyzed in various NK cell subsets based on their CD56, CD57, NKG2A, and KIR expression. CD107a and IFNγ expression were indicated either as absolute values **(G)** or as the percentage decrease **(A–F,H)** due to MTA co-incubation [(absolute value with MTA/absolute value without MTA)*100]. Significance was calculated using a Wilcoxon test for analyzing paired samples **(A,B,G,H)** or a one-way ANOVA **(C–F)** test (*p* value: * <0.05; *n* = 12).

Next, we aimed to analyze the suppressive MTA effects within the different NK cell subsets. We first analyzed the MTA-induced reduction of CD107a and IFNγ expression upon K562 cell stimulation within CD56^bright^ and CD56^dim^ NK cells. While the suppressive MTA effect on NK cell degranulation was similar between both subsets (Figure 4A), the effect on IFNγ production was significantly more pronounced within the CD56^dim^ subset (Figure 4B). Recently, it has been demonstrated that the CD56^dim^ subset can be further divided into functional distinct subsets based on the expression of NKG2A, KIR, and CD57 (15). In terms of NK cell degranulation, the suppressive MTA effect was similar between the distinct subsets, independent if K562 cells (Figure 4C) or CD16 antibodies (Figure 4E) were used for stimulation. The same was observed for IFNγ production upon K562 cell stimulation (Figure 4D). In contrast, IFNγ production upon CD16 stimulation was less reduced in CD56^dim^ NK cells expressing CD57 on their surface (Figure 4F). CD57^+^ NK cells produced significantly more IFNγ upon CD16 (Figure 4G) or K562 stimulation (Supplementary Figure 4B) than CD57^−^ ones, while no difference in CD107a expression was observed (Supplementary Figures 4A,C). Interestingly, IFNγ production upon CD16 stimulation was significantly less effected by MTA in CD57^+^ than CD57^−^ NK cells (Figure 4H), while no difference was observed upon K562 cell stimulation (Supplementary Figure 4E) and for CD107a expression (Supplementary Figures 4D,F). These differences in IFNγ production upon CD16 stimulation could be due to the significantly higher CD16 expression levels on CD57^+^ NK cells resulting in a stronger activation of this NK cell subset (Supplementary Figure 4G). In summary, the suppressive MTA effect on IFNγ production is present in all studied NK subsets with varying extent between the distinct NK cell subsets; CD57^+^ NK cells in particular seem less sensitive than CD57^−^ NKs.

### Gene Expression of MTA's Metabolic Pathway and Targets Is Similar in CD57^+/−^ NK Cells

**Figure 5:**
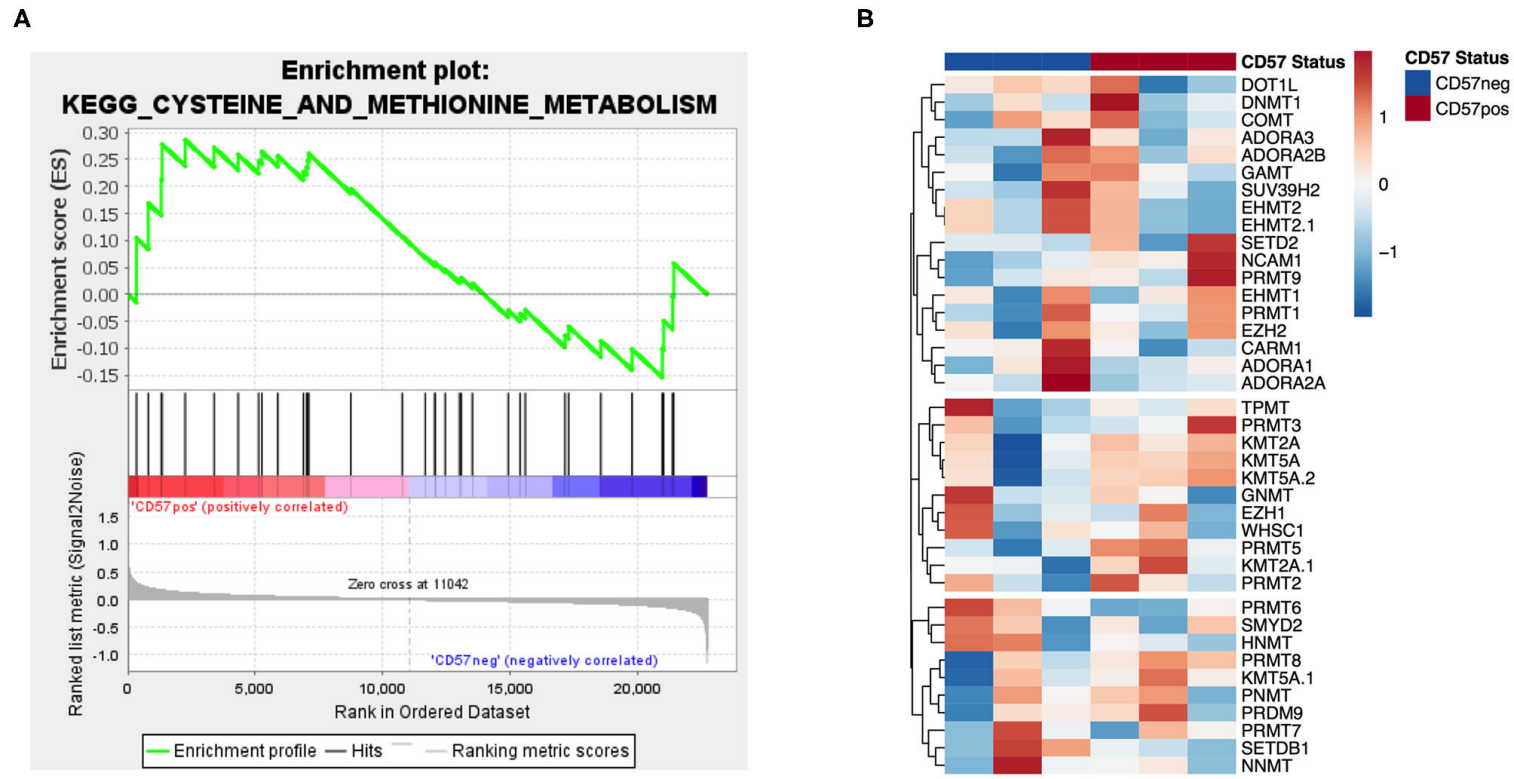
Gene expression of MTA's metabolic pathway and targets is similar in CD57^+/−^ NK cells. Analysis of publicly available microarray data (GSE23695) (20) of CD56^dim^CD16^+^CD57^+^ and CD57^−^ sorted NK cells performing a gene set enrichment analysis of the methionine and cysteine metabolism pathway **(A)** and a differential gene expression analysis of adenosine receptors and various methyltransferases **(B)**. For the gene set enrichment analysis, the normalized enrichment score (NES) was 0.8 and the nominal *p* value 0.6.

In order to identify potential mechanisms for the differential MTA susceptibility of the two subsets, we analyzed the publicly available microarray dataset GSE23695 of CD56^dim^CD16^+^CD57^+^ and CD57^−^ sorted NK cells (20). A GSEA of the methionine and cysteine pathway, including MTAP as the first degradation step of MTA, revealed no significant enrichment in any of the two subsets (Figure 5A). In addition, none of the four known adenosine receptors nor any of the most important methyl transferases, including PRMT5, were differentially expressed between the two groups (Figure 5B and Supplementary Table 1). All in all, CD57^+^ and CD57^−^ NK cells do not differ in their gene expression profile concerning known MTA targets or enzymes of the methionine (MTA) metabolic pathway.

### Donors Haboring a NKG2C^+^ NK Cell Expansion Are Less Sensitive Against MTA-Induced Suppression of IFNγ Production

**Figure 6:**
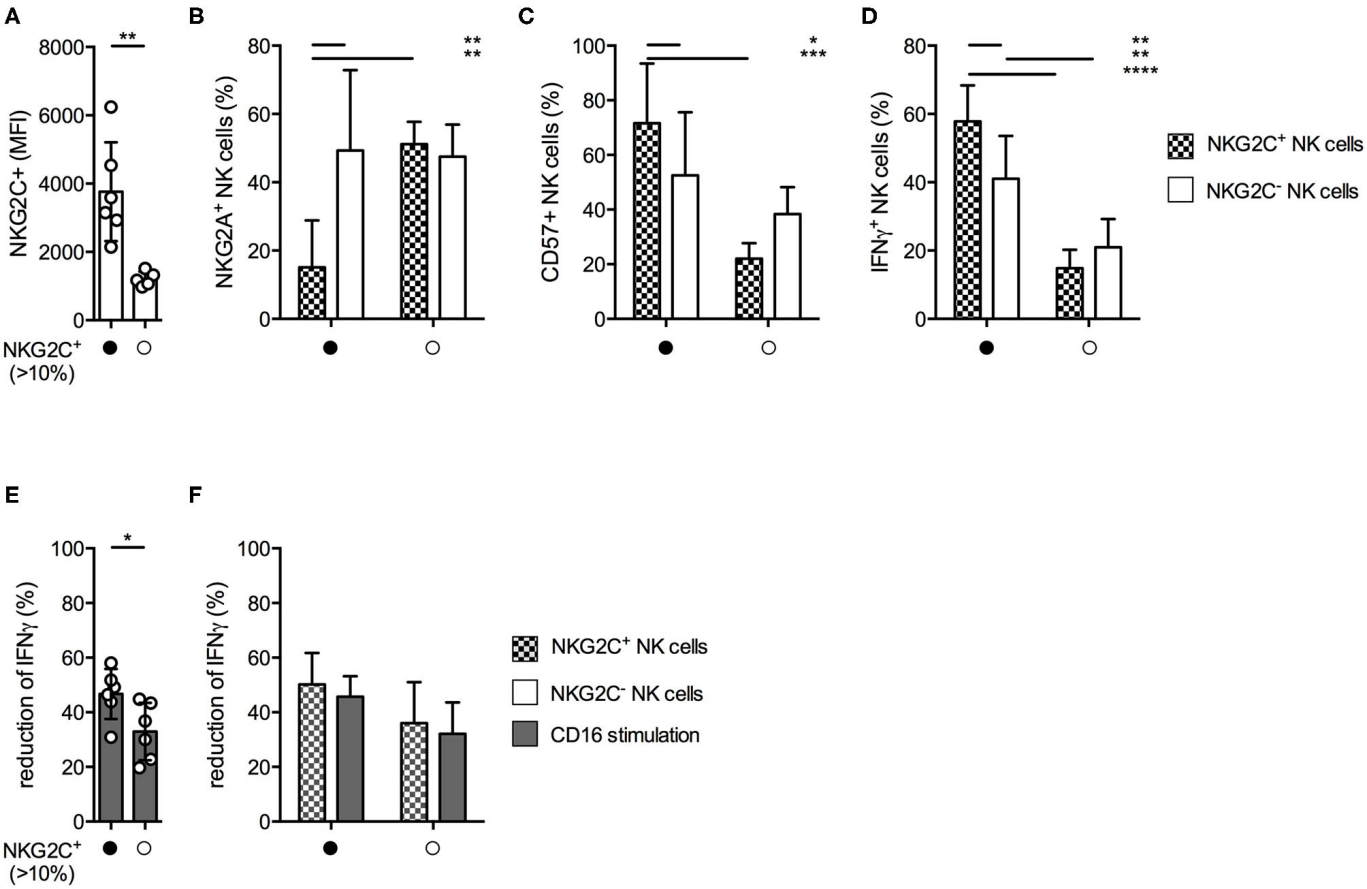
MTA-induced suppression of IFNγ production is less pronounced in donors harboring an expansion of NKG2C^+^ NK cells. Healthy donors were divided into two groups based on the presence of an expansion of NKG2C^+^ expressing NK cells (>10%). Overnight IL-2-activated NK cells were stained for the surface expression of NKG2C, NKG2A, and CD57 **(A–C)** or pre-incubated with 100 μM MTA for 30′ at 37°C and then stimulated with plate-bound CD16 for additional 4 h at 37°C **(D–F)**. Afterwards, the cells were harvested and IFNγ expression was analyzed in NKG2C^+/−^ NK cells. IFNγ expression was indicated either as absolute values **(D)** or as the percentage decrease **(E,F)** due to MTA co-incubation [(absolute value with MTA/absolute value without MTA)*100]. Significance was calculated using a Mann–Whitney test for analyzing unpaired samples **(A,E)** or a two-way ANOVA **(B–D,F)** test (*p* value: * < 0.05, ** < 0.01, *** < 0.001, **** < 0.0001, *n* = 6).

Since we observed varying degrees of MTA-induced suppression of IFNγ production within the different NK cell subsets, we were interested if similar effects could be observed within the NKG2C^+^ NK cell subset. This highly differentiated subset typically expands during CMV infection, demonstrates long-term survival, has increased capacity of IFNγ production, and is transplantable (24, 25). We identified 6 out of 12 healthy donors with an expansion of NKGC2^+^ NK cells, defined as >10% of total NK cells. Their NKG2C surface density was significantly higher compared to those donors without a NKG2C^+^ expansion (Figure 6A). In accordance with other reports (25) these NKG2C^hi^ NK cells expressed significantly less NKG2A (Figure 6B) and higher levels of CD57 (Figure 6C) on their surface than their NKG2C^−^ counterparts within the same donor, as well as compared to NKG2C^+^ NK cells from donors without a NKG2C^+^ expansion. In addition, NKG2C^hi^ NK cells demonstrated superior IFNγ production upon CD16 stimulation (Figure 6D). NK cells from donors exhibiting a NKG2C^+^ expansion were less susceptible toward MTA-induced suppression of IFNγ production upon CD16 stimulation than NK cells from donors without one (Figure 6E). However, when comparing the suppressive MTA effect between NKG2C^+/−^ NK cells in both donor groups, no significant differences were observed (Figure 6F). Interestingly, independent of their NKG2C expression, all NK cells from donors harboring a NKG2C^+^ expansion tend to be less susceptible toward MTA-induced suppression compared to those without an expansion. In addition, no differences between donors with or without a NKG2C^+^ expansion were observed for K562-induced IFNγ production (Supplementary Figure 5B) or CD107a expression independent of the used stimuli (Supplementary Figures 5A,C). Moreover, no significant differences in CD16 expression levels were observed between these two groups of donors (Supplementary Figure 5D). Overall, we observed a reduced susceptibility toward MTA-induced suppression of IFNγ production upon CD16 stimulation in donors harboring a NKG2C^+^ expansion. Strikingly, this effect was not due to a reduced susceptibility of NKG2C^+^ NK cells toward MTA compared to NKG2C^−^ ones, but rather donor dependent.

### Conjugate Formation and Inside-Out Signaling Is Reduced by MTA

**Figure 7:**
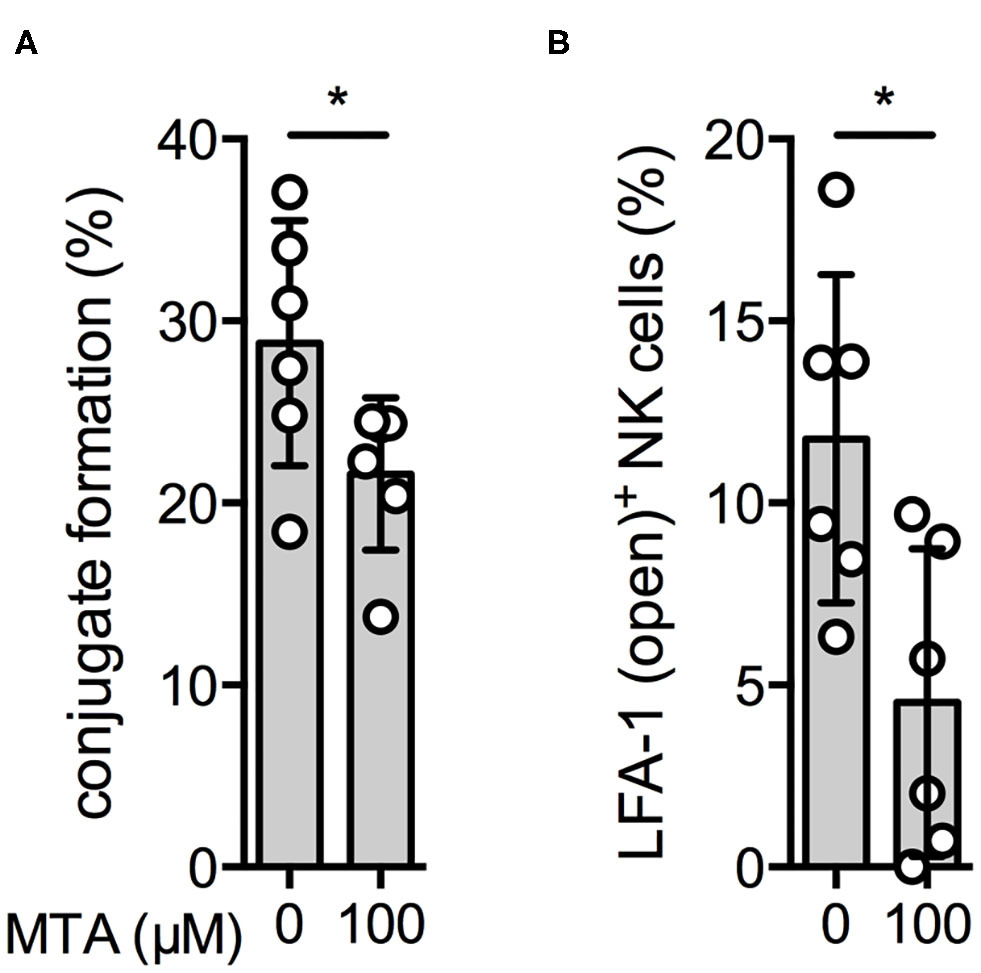
Conjugate formation and inside-out signaling are reduced by MTA. Overnight IL-2-activated NK cells (VPD450) and K562 cells (PKH-26) were labeled with different fluorescent dyes. NK cells were pre-incubated with or without 100 μM MTA and afterwards co-incubated with K562 cells for 10′. Conjugate formation was calculated as the percentage of double positive events (VPD450/PKH-26 positive cells) of all NK cells (VPD450 positive cells) **(A)**. In addition, expression of the open conformation LFA-1 isoform on NK cells was analyzed as well **(B)**. Significance was calculated using a Wilcoxon test for analyzing unpaired samples (*p* value: * <0.05; *n* = 6).

Our next aim was to investigate if MTA is already inhibiting early events during NK cell activation, ultimately leading to a reduced NK cell degranulation and IFNγ production. We first tested if MTA reduces the amount of conjugate formations between NK and K562 cells during their initial contact. In the presence of 100 μM MTA, NK:K562 cell conjugate formation was significantly reduced (Figure 7A).

Another early event during NK cell activation is the formation of the so-called inside-out signal. It is attributed to the conformation change of LFA-1, an adhesion molecule, upon NK cell activation resulting in the “open-conformation” LFA-1 isoform, which has a higher binding affinity and increases the NK cell's attachment to its target cell (26). In the presence of MTA, expression of the LFA-1 open conformation isoform upon K562 cell stimulation was significantly reduced (Figure 7B). Altogether, we demonstrate that MTA suppresses early events during NK cell activation, like conjugate formation and inside-out signaling.

### MTA Inhibits the NF-κB, PI3K/AKT/S6, and MAPK/ERK Pathways Downstream of the CD16 Receptor

**Figure 8:**
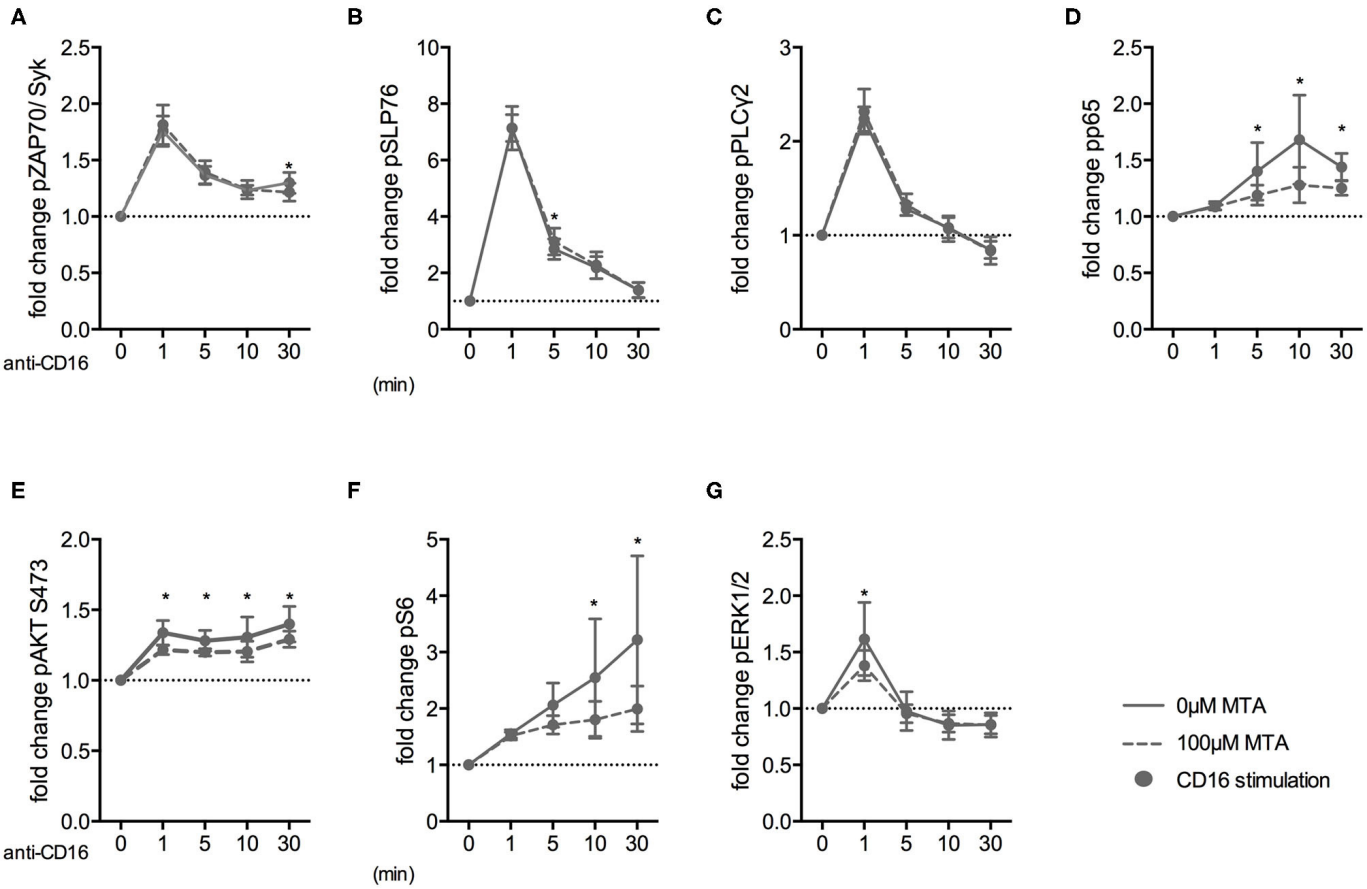
MTA inhibits the NF-κB, PI3K/AKT/S6, and MAPK/ERK pathways downstream of the CD16 receptor. Freshly isolated NK cells were pre-incubated with or without 100 μM MTA for 30′ at 37°C and then stimulated with anti-CD16 antibodies for 0, 1, 5, 10, and 30′. Cells were labeled with a fluorescent bar cell code and stained for various phospho-epitopes of the CD16 signaling cascade. The fold increase of phospho-epitope staining was calculated for the different time points compared to baseline staining at 0′ (**A–G**; *n* = 6). Significance was calculated using a two-way ANOVA test (*p* value: * <0.05).

Finally, we investigated how early the suppressive MTA effect on NK cells was evident during their activation. Therefore, we analyzed the expression of various phospho-epitopes upon CD16 stimulation in the presence or absence of 100 μM MTA. Freshly isolated NK cells were stimulated for up to 60′ and subsequently analyzed using fluorescent cell barcoding and phospho-flow technologies (Supplementary Figures 6A,B). Interestingly, MTA had no effect on the phosphorylation of the proximal signaling molecules ZAP70/Syk upon CD16 stimulation (Figure 8A). Similarly, phosphorylation of SLP76 (Figure 8B) and PLCγ2 (Figure 8C) remained mostly unaffected by MTA co-incubation. However, the NF-κ*B* (Figure 8D), PI3K/AKT/mTOR (Figures 8E,F) and MAPK/ERK (Figure 8G) pathways, which are involved in NK cell degranulation and cytokine production, were all negatively influenced by MTA. Additionally, we further investigated the negative MTA effect on the PI3K/AKT/mTOR pathway in CD57^+^ and CD57^−^ NK cells. We observed a tendency that S6 phosphorylation upon CD16 stimulation was stronger inhibited by MTA in CD57^−^ than in CD57^+^ NK cells (Supplementary Figure 6C). Together, these data demonstrate that the suppressive MTA effect on the cytotoxic activity of NK cells is due to its early inhibitory effect on various signaling pathways of activating NK cell receptors.

## Discussion

Loss of MTAP expression in tumor cells results in the accumulation of MTA (27). MTAP deficiency has been reported for several tumor entities, with high frequency in mesothelioma, T cell acute lymphoblastic leukemia (T-ALL), gliomas, metastatic melanoma, and non-small cell lung cancer (NSCLC) (28). It has been demonstrated that MTAP-deficient tumors exhibit a more aggressive clinical course with increased proliferation, invasiveness, and metastasis (29). In NSCLC, loss of MTAP is associated with poor overall and disease-free survival (30). In addition, MTAP deficiency is a predictive marker for the response to adjuvant interferon therapy in melanoma patients (31). However, to the best of our knowledge, no data are available on NK cell numbers and functions in these entities stratified by MTAP expression levels.

In the current study, we demonstrated that MTA suppresses several NK cell functions, which is in line with observations in other immune cells. Our group discovered that MTA blocks T cell metabolism and proliferation, thereby suppressing the induction of antigen-specific CD8 T cells and cytokine production by CD4 T cells. Interestingly, the MTA effect was reversible since T cells regained their proliferative capacity after removal of MTA from the culture medium (7, 8). Moreover, TNF production in macrophages upon toll-like receptor (TLR) stimulation, e.g., with LPS, is sufficiently suppressed by MTA in an adenosine A2 receptor-dependent manner (9, 10). These results have been demonstrated successfully in a mouse model *in vivo* where LPS-induced lethality was completely prevented by MTA injection due to reduced TNF and increased IL-10 levels (32). Similar results have been observed in an autoimmune encephalomyelitis (EAE) and a colon cancer mouse model, in which MTA treatment prevented acute EAE and reduced the inflammation-induced tumor load in treated mice, respectively (33, 34). Overall, these results clearly demonstrate a suppressive effect of MTA on innate and adaptive immune cells.

However, an important question is whether the used MTA concentrations are physiological. MTA concentrations within MTAP-deficient human melanoma cell lines have been reported to be up to 140 nM (29) and up to 1,000 nM within the supernatant (7). However, due to the close proximity and narrow space between a tumor and NK cell during immunological synapse formation, effective *in situ* MTA concentrations are to be expected significantly higher than those measured under *in vitro* cell culture conditions. We used a MTA concentration of 100 μM in our experiments, but also observed a suppressive effect on NK cells at lower concentrations. This is in line with the MTA concentration that has been used in other publications that investigated MTA's suppressive effect on immune cells *in vivo* and *in vitro* (7, 9–11, 35).

MTA is known to execute its effect by different mechanisms. In melanoma cells, MTA activates the transcription factor AP-1, potentially leading to increased expression of metalloproteases and growth factors (29). The activation is dependent on the adenosine receptor ADORA2B, since blocking or knockdown of the receptor resulted in reduced AP-1 signaling. Interestingly, MTA did not cause an increase in cyclic adenosine monophosphate (cAMP) or intracellular calcium levels (21). This is in line with previous reports demonstrating that incubation with MTA concentrations ≥100 μM did not cause an increase of cAMP levels in human peripheral lymphocytes, whereas NK cell cytotoxicity was significantly reduced at these concentrations, confirming our observations (11).

Importantly, MTA led to an accumulation of S-adenosyl-homocysteine (AdoHcy), due to its inhibitory effect on the AdoHcy hydrolase (36, 37). AdoHcy is a by-product during methylation reactions involving S-adenosyl-methionine (AdoMet) as a methyl-group donor, and, if not properly removed, inhibits methylation reactions (38, 39). The role of MTA in interfering in protein methylation processes has been proposed for a long time (40) and, besides its indirect effect *via* inhibiting AdoHcy hydrolase, recent works demonstrated a direct inhibition of several protein arginine methyltransferases (PRMT). MTA selectively inhibits PRMT5 at low concentrations (≤ 10 μM), whereas other PRMTs were inhibited only at higher concentrations. MTAP-deficient tumor cells accumulate high levels of MTA leading to a reduced PRMT5 activity, which renders these cells more susceptible toward additional PRMT5 inhibition compared to MTAP-competent cells (8, 22, 41, 42). Cell signaling events are mediated by different protein–protein interactions, which are regulated through post-translational modification (PTM) of which phosphorylation is the best studied one (43). However, in recent years, modification of proteins by methylation during signal transduction has stepped into the spotlight of research. We revealed that distinct signaling pathways downstream of the CD16 receptors were suppressed by MTA. Several groups demonstrated that MTA is able to suppress the PI3K/AKT/mTOR axis by reducing AKT and S6 phosphorylation (7, 8, 44), which is in line with our observation. Interestingly, a current work demonstrated that, upon activation, AKT is methylated by the histone methyltransferase SETDB1, resulting in sustained AKT phosphorylation and signaling (45). In addition, the NF-κB pathway is known as well to be regulated by methylation of arginine and lysine residues of the p65 subunit of NF-κB (46). Upon activation with IL-1β, PRMT5 dimethylates arginine 30 (R30) of the p65 subunit, leading to increased expression of NF-κB-induced genes (47). Although we did not observe a suppressive MTA effect on SLP-76 or PLCγ2 phosphorylation, another member of this signaling pathway, Vav-1, is known to be regulated by protein methylation. Upon CD28 engagement, Vav-1 was R-methylated, which could be blocked by incubation with 300 μM MTA (35). In summary, our results indicate that MTA blocks phosphorylation and methylation of distinct signaling pathways downstream of activating NK cell receptors by its suppressive effect on protein methylation rather than by the suppressive ability of its adenosine residue.

Here, we discovered that MTA blocks NK cell effector functions by interfering with NK activation to the end that IFNγ production was significantly stronger affected than degranulation. It has been demonstrated that signals of different strengths are needed for the individual NK cell functions including degranulation, chemokine, and cytokine production. While the signal of a single activating receptor is sufficient to induce chemokine production, NK cell degranulation needs the coordinated engagement of two activating receptors. Moreover, IFNγ production depends on the activation of several receptors, pointing to high regulatory demands for sufficient IFNγ production and therefore to a higher susceptibility toward MTA suppression (48, 49).

In addition, we observed that IFNγ production upon CD16 stimulation was significantly higher in CD57-expressing NK cells and less susceptible toward MTA suppression. This is in line with previous reports demonstrating that during terminal differentiation, which is accompanied by CD57 expression, NK cells produced higher levels of IFNγ upon activation. Mechanistically, terminal differentiated NK cells show a higher NF-κB activation upon stimulation, higher *IFNG* and *TBX21* transcripts, as well as demethylation of the *IFNG* promoter (50). Given the fact that MTA reduces NF-κB signaling in NK cells upon CD16 engagement, it is likely that the stronger NF-κB activation in CD57^+^ NK cells upon activation is sufficient to partially overcome MTA suppression. This is further supported by our observation of a stronger MTA-induced inhibition of S6 phosphorylation upon CD16 stimulation in CD57^−^ than in CD57^+^ NK cells, since the PI3K/AKT/mTOR pathway contributes to IFNγ production in NK cells as well (51). Importantly, we observed differences within the CD16 expression levels between these two subsets, an indication that CD57^+^ are probably receiving a stronger initial activation signal upon CD16 stimulation, which is sufficient to partly overcome MTA suppression. In addition, the data from the microarray analysis between CD57^+^ and CD57^−^ NK cells demonstrate that neither the expression of adenosine receptors, various methyltransferases (including PRMT5), nor enzymes of the methionine salvage pathway (including MTAP) differ between those two subsets.

Furthermore, IFNγ production was less affected by MTA suppression in healthy donors, harboring a NKG2C^+^ expansion. These NKG2C^+^ cells have been demonstrated to be major IFNγ producers upon CD16 stimulation due to epigenetic modulation of their *IFNG* locus (52). Additionally, CD16-induced phosphorylation of signaling molecules like SLP-76, ERK1/2, and S6RP was stronger in NKG2C^+^ NK cells compared to NKG2C^−^ ones (25).

Surprisingly, when comparing NKGC2^+/−^ NK cells in donors with a NKG2C^+^ expansion with each other, we only detected a small, non-significant difference in the susceptibility toward MTA. Moreover, NKG2C^−^ NK cells of NKG2C^+^ expansion donors demonstrated superior IFNγ production compared to NKG2C^+/−^ NK cells in donors without an expansion. Therefore, the difference between the two donor groups might rely not only on the existence of a NKG2C^+^ expansion but also on the whole NK cell population. One explanation could be the higher frequency of CD57-expressing NK cells within NKG2C^+^ and NKG2C^−^ NK cells compared to their counterparts in donors without an expansion. Another effect could be that our group of NKG2C^+^ expansion donors are harboring a polymorphism of their CD16 receptor resulting in a higher affinity to human IgG1 and therefore superior activation upon stimulation (53). Importantly, no significant differences were observed within the CD16 expression levels of these two groups of donors. All in all, we observed differences in MTA-induced suppression of distinct NK cell functions and subsets, which could be explained due to differences in the strength of activating signaling pathways within the various subsets upon stimulation.

Overall, the current work demonstrates additional mechanisms by which tumor cells are able to escape NK cell surveillance, illustrating the necessity for new strategies to counteract these escape mechanisms. MTAP-deficient tumor cells are more susceptible toward PRMT5 inhibition than MTAP-competent ones, so using PRMT5 inhibitors is an opportunity to specifically target this tumor cell group (22, 41, 42). Although we demonstrated that PRMT5 inhibition is also able to reduce NK cell degranulation, a very high concentration of the inhibitor was needed to observe an effect. Based on our results, another opportunity to overcome MTA suppression is to increase the strength of the activation signal in NK cells. In the case of adoptive NK cell transfer treatments, a possibility would be to increase the percentage of CD57-expressing NK cells within the cell product as we demonstrated that these cells were less susceptible toward MTA suppression than CD57^−^ ones. This could be achieved, for example, by expanding NKG2C^+^ NK cells from donors harboring a NKG2C^+^ expansion, as these cells express high levels of CD57. Liu et al. demonstrated that specific expansion of NKG2C^+^ NK cells is feasible by stimulating NK cells with HLA-E, the ligand for NKG2C, and IL-15. NKG2C^+^ NK cells expanded within 14 days, expressed high levels of CD57, and demonstrated superior effector cell functions against PHA and pediatric ALL blasts (54). In addition, genetic manipulation of these NK cells to overexpress MTAP before using them for adoptive cell transfer could prove beneficial.

## Data Availability Statement

The datasets generated for this study are available on request to the corresponding author.

## Ethics Statement

The studies involving human participants were reviewed and approved by Local Ethics committee of the University of Erlangen and Oslo. The patients/participants provided their written informed consent to participate in this study.

## Author Contributions

BJ, AM, and MA contributed to the conception and design of the study. DM advised on the topic of metabolomics. SV advised on the topic of phospho-epitope staining. K-JM advised on the topic of NK cell subsets and adaptive NK cells. BJ, SS, CS, and AS performed the experiments and the statistical analysis. BJ wrote the first draft of the manuscript. MA revised and wrote sections of the manuscript. All authors contributed to the article and approved the submitted version.

## Conflict of Interest

The authors declare that the research was conducted in the absence of any commercial or financial relationships that could be construed as a potential conflict of interest.

## Supplementary Material

Supplementary Figure 1 NK cells' cytotoxic activity is reduced upon MTA co-incubation without affecting NK cell viability. Isolated NK cells were incubated overnight with 100 U/ml IL-2 and then incubated for 4 h with various concentrations of MTA at 37°C. NK cells were then harvested and stained for 7AAD expression (**A**; *n*: 8). Significance was calculated using a one-way ANOVA test.

Supplementary Figure 2 NK cell degranulation and cytokine production is reduced by MTA. Overnight IL-2 activated NK cells were pre-incubated with different concentrations of MTA for 30′ at 37°C and then either stimulated with K562 cells at a 1:1 ratio (**A**; *n*: 6) or left alone (**B**; *n*: 12) with the prior MTA concentration for additional 4 h at 37°C. Afterwards the cells were harvested and CD107a **(A)** or CD16 **(B)** expression were analyzed in bulk NK cells. Significance was calculated using a Wilcoxon test for analyzing paired samples (*p*-value: ^*^ < 0.05).

Supplementary Figure 3 Adenosine derivates and PRMT5 inhibitor reduce NK cell function similar to MTA. Overnight IL-2 activated NK cells were pre-incubated for 30′ with 50μM 3-deazaadenosine (3-Deaza; **A**) or 5-azacitidine (5-Aza; **B**) and then stimulated with K562 cells at a 1:1 ratio for additional 4 h at 37°C (*n*: 6). Afterwards the cells were harvested and CD107a expression was analyzed in bulk NK cells. Significance was calculated using a Wilcoxon test for analyzing paired samples (*p*-value: ^*^ < 0.05).

Supplementary Figure 4 MTA's inhibitory effect varies within the different NK cell subsets. Overnight IL-2 activated NK cells were stimulated with K562 cells at a 1:1 ratio **(A,B,D,E)** or plate-bound CD16 **(C,F)** for 4h at 37°C with **(D–F)** or without **(A–C)** 100μM MTA. Afterwards the cells were harvested and CD107a or IFNγ expression was analyzed in NK cell subsets based on their CD57 expression. CD107a and IFNγ expression were indicated either as absolute values **(A–C)** or as the percentage decrease **(D–F)** due to MTA co-incubation [(absolute value with MTA/ absolute value without MTA)^*^100]. CD16 expression levels (MFI) were analyzed on CD57+/− NK cells **(G)**. Significance was calculated using a Wilcoxon test for analyzing paired samples (*p*-value: ^*^ < 0.05, ^****^ < 0, 0001; *n*: 12).

Supplementary Figure 5 MTA-induced suppression of IFNγ production is less pronounced in donors harboring an expansion of NKG2C^+^ NK cells. Healthy donors were divided into two groups based on the presence of an expansion of NKG2C expressing NK cells (>10%). Overnight IL-2 activated NK cells were pre-incubated with 100μM MTA for 30′ at 37°C and then stimulated with K562 cells at a 1:1 ratio **(A,B)** or with plate-bound CD16 **(C)** for additional 4 h at 37°C. Afterwards the cells were harvested and CD107a and IFNγ expression were analyzed in bulk NK cells. CD107a and IFNγ expression were indicated as the percentage decrease due to MTA co-incubation [(absolute value with MTA/ absolute value without MTA)^*^100]. CD16 expression levels (MFI) were analyzed on CD56^dim^ NK cells within the two donor groups **(D)**. Significance was calculated using a Mann-Whitney test for analyzing unpaired samples (*n*: 6).

Supplementary Figure 6 MTA inhibits the NF-κB, PI3K/AKT/S6 and MAPK/ERK pathways down-stream of the CD16 receptor. Freshly isolated NK cells were pre-incubated with or without 100 μM MTA for 30′ at 37°C and then stimulated with anti-CD16 antibodies for 0, 1, 5, 10, 30, and 60′. Bulk **(A,B)** or CD57+/- NK cells **(C)** were labeled with a fluorescent bar cell code **(A)** and stained for the phospho-epitope S6 (**B,C**, *n*:5).

Supplementary Table 1 Differential expression of adenosine receptors and methyltransferases in CD57^+/−^ NK cells.
