# Supplementary figures and images for: The Oncometabolite 5′-Deoxy-5′-Methylthioadenosine Blocks Multiple Signaling Pathways of NK Cell Activation
Source: Front Immunol. 2020 Oct 6;11:2128. doi: 10.3389/fimmu.2020.02128 (PMC7573074; doi:10.3389/fimmu.2020.02128)

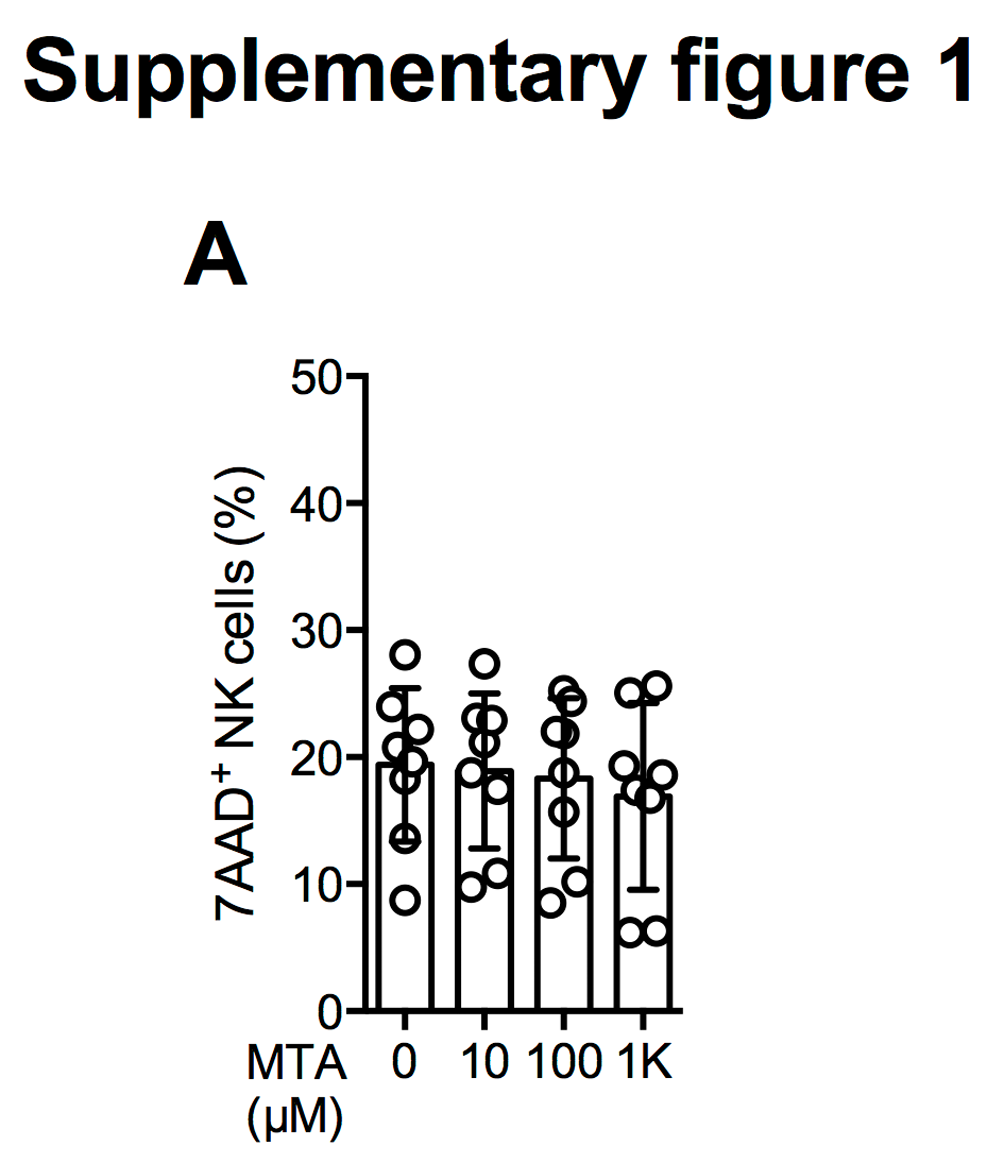

Supplement: Supplementary Figure 1 — NK cells' cytotoxic activity is reduced upon MTA co-incubation without affecting NK cell viability. Isolated NK cells were incubated overnight with 100 U/ml IL-2 and then incubated for 4 h with various concentrations of MTA at 37°C. NK cells were then harvested and stained for 7AAD expression (A; n: 8). Significance was calculated using a one-way ANOVA test. [file Image_1.TIFF]

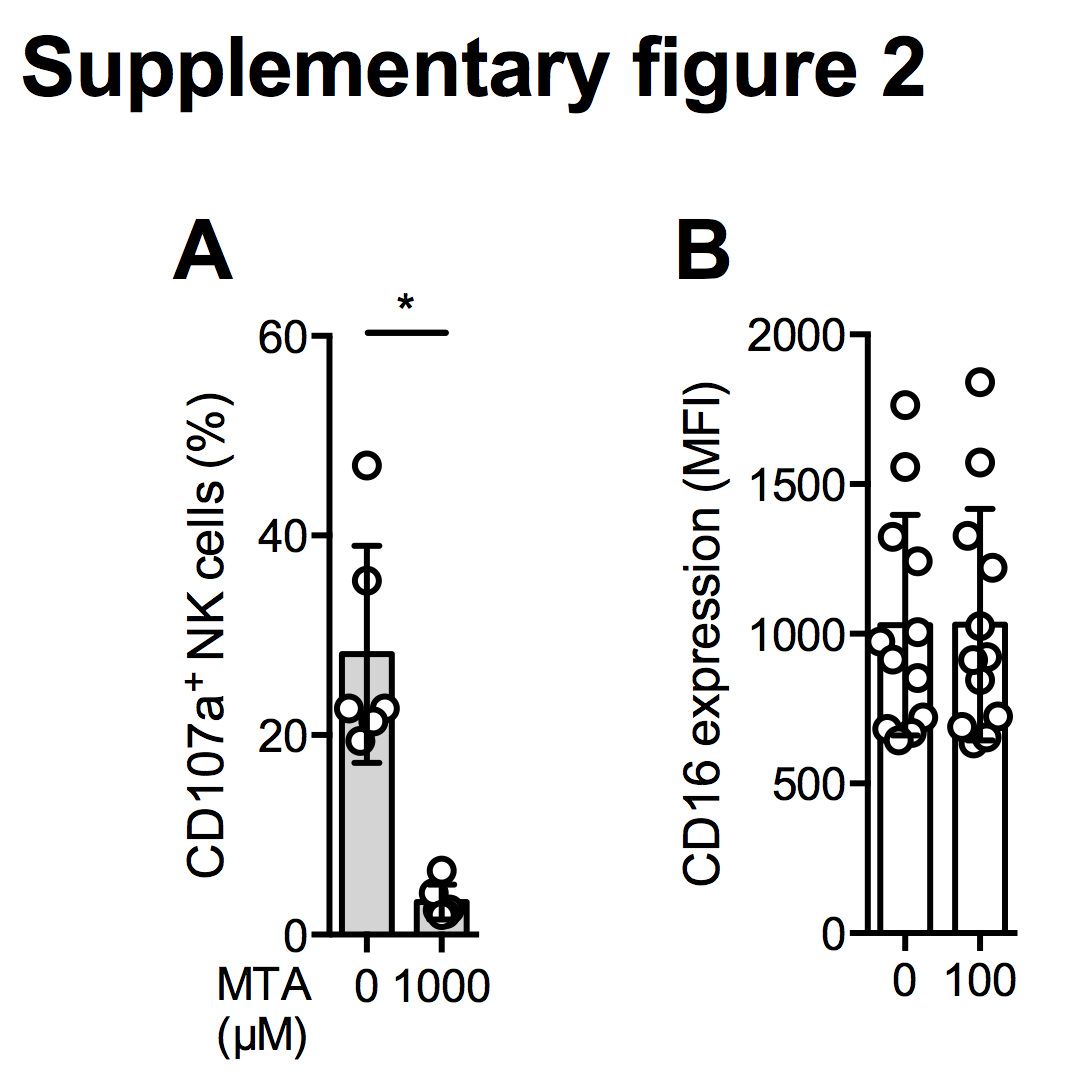

Supplement: Supplementary Figure 2 — NK cell degranulation and cytokine production is reduced by MTA. Overnight IL-2 activated NK cells were pre-incubated with different concentrations of MTA for 30′ at 37°C and then either stimulated with K562 cells at a 1:1 ratio (A; n: 6) or left alone (B; n: 12) with the prior MTA concentration for additional 4 h at 37°C. Afterwards the cells were harvested and CD107a (A) or CD16 (B) expression were analyzed in bulk NK cells. Significance was calculated using a Wilcoxon test for analyzing paired samples (p-value: * < 0.05). [file Image_2.TIFF]

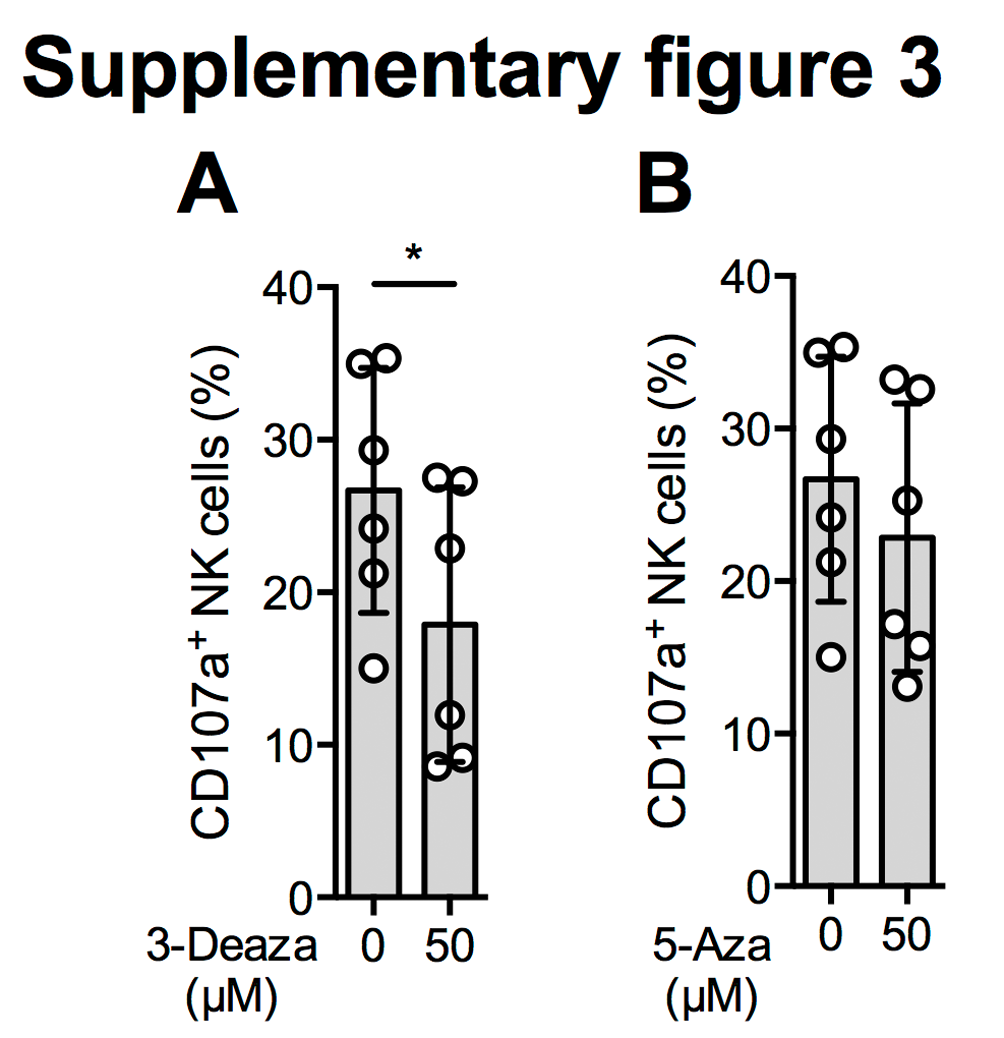

Supplement: Supplementary Figure 3 — Adenosine derivates and PRMT5 inhibitor reduce NK cell function similar to MTA. Overnight IL-2 activated NK cells were pre-incubated for 30′ with 50μM 3-deazaadenosine (3-Deaza; A) or 5-azacitidine (5-Aza; B) and then stimulated with K562 cells at a 1:1 ratio for additional 4 h at 37°C (n: 6). Afterwards the cells were harvested and CD107a expression was analyzed in bulk NK cells. Significance was calculated using a Wilcoxon test for analyzing paired samples (p-value: * < 0.05). [file Image_3.TIFF]

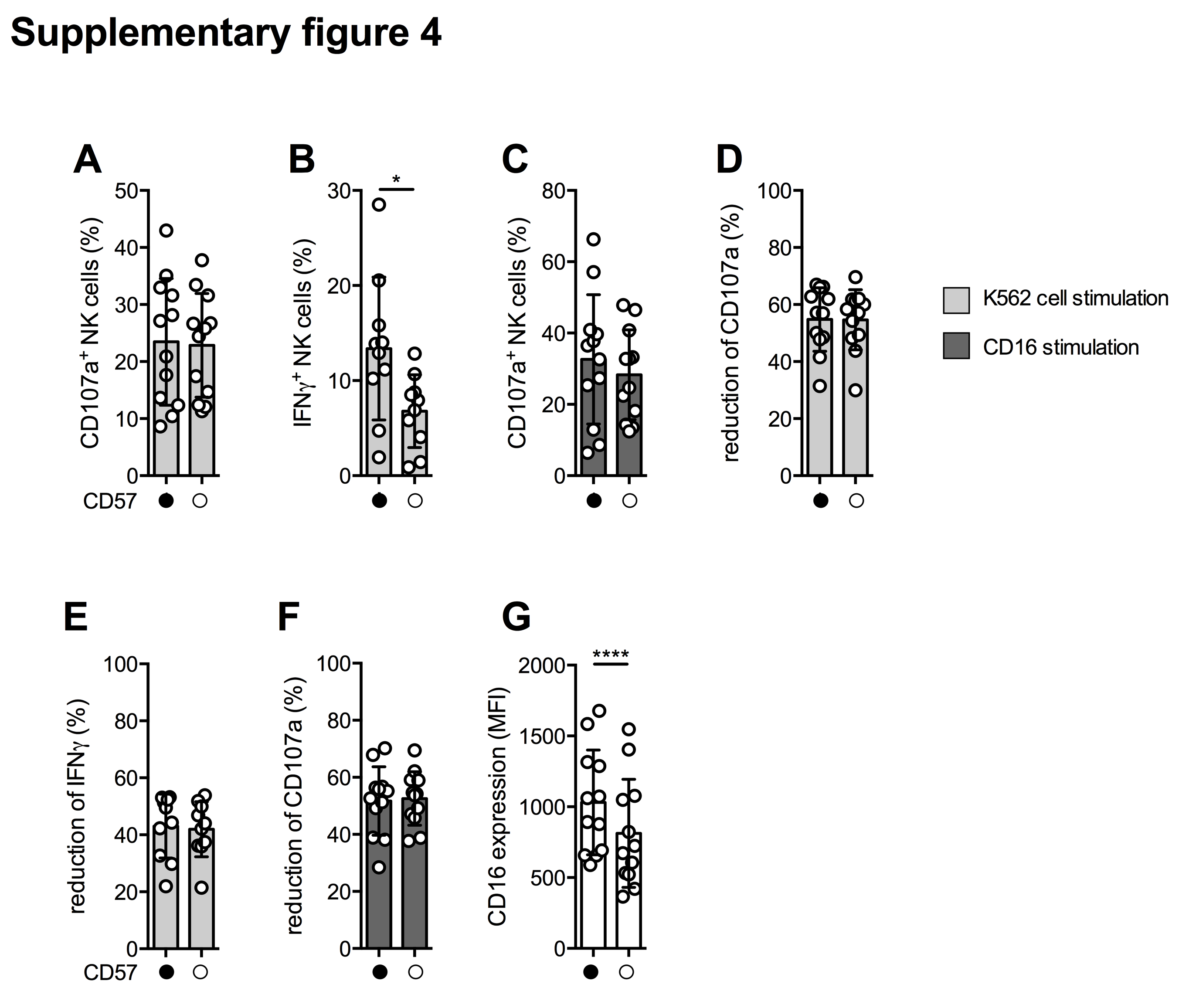

Supplement: Supplementary Figure 4 — MTA's inhibitory effect varies within the different NK cell subsets. Overnight IL-2 activated NK cells were stimulated with K562 cells at a 1:1 ratio (A,B,D,E) or plate-bound CD16 (C,F) for 4h at 37°C with (D–F) or without (A–C) 100μM MTA. Afterwards the cells were harvested and CD107a or IFNγ expression was analyzed in NK cell subsets based on their CD57 expression. CD107a and IFNγ expression were indicated either as absolute values (A–C) or as the percentage decrease (D–F) due to MTA co-incubation [(absolute value with MTA/ absolute value without MTA)*100]. CD16 expression levels (MFI) were analyzed on CD57+/− NK cells (G). Significance was calculated using a Wilcoxon test for analyzing paired samples (p-value: * < 0.05, **** < 0, 0001; n: 12). [file Image_4.TIFF]

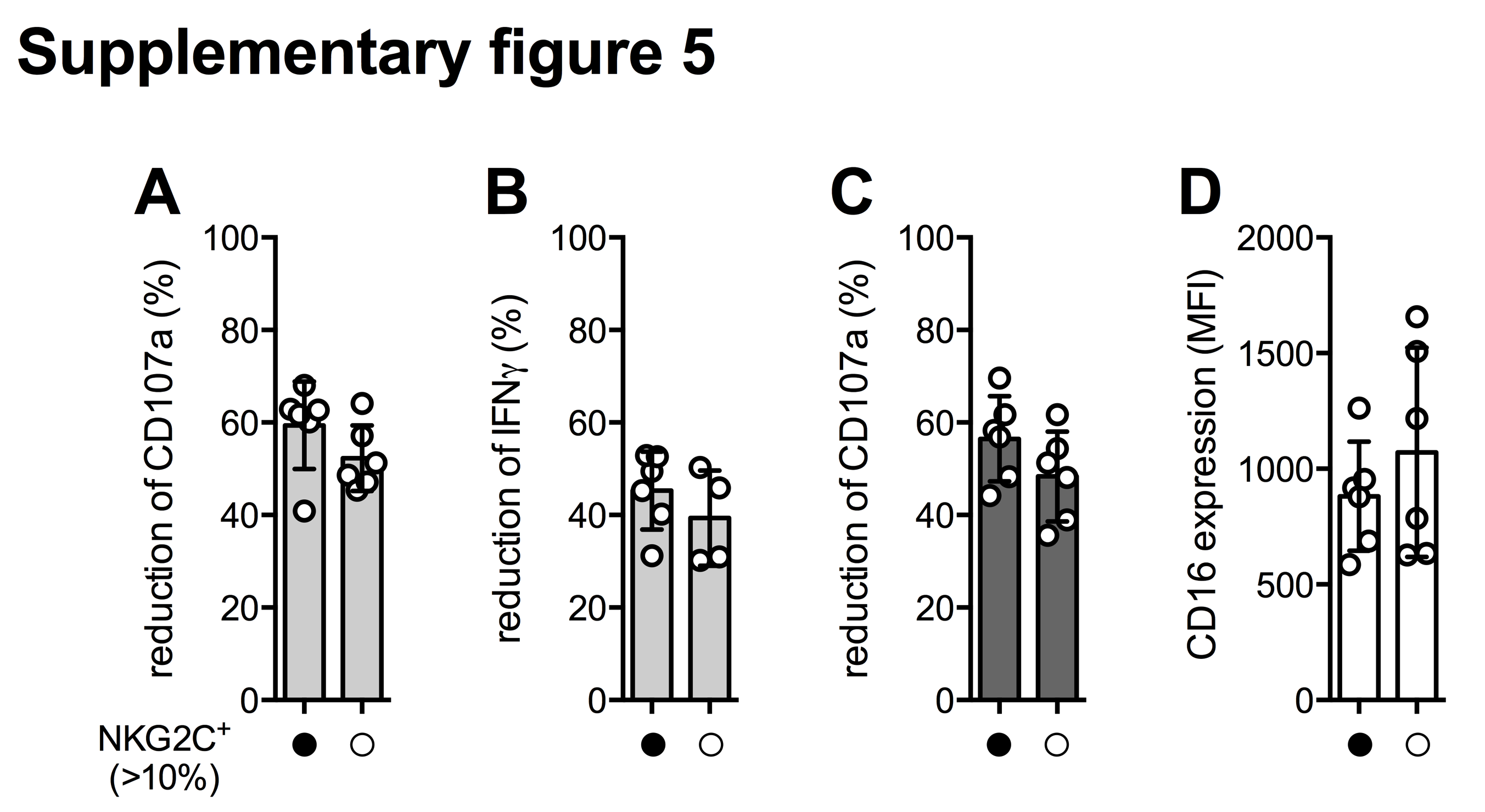

Supplement: Supplementary Figure 5 — MTA-induced suppression of IFNγ production is less pronounced in donors harboring an expansion of NKG2C+ NK cells. Healthy donors were divided into two groups based on the presence of an expansion of NKG2C expressing NK cells (>10%). Overnight IL-2 activated NK cells were pre-incubated with 100μM MTA for 30′ at 37°C and then stimulated with K562 cells at a 1:1 ratio (A,B) or with plate-bound CD16 (C) for additional 4 h at 37°C. Afterwards the cells were harvested and CD107a and IFNγ expression were analyzed in bulk NK cells. CD107a and IFNγ expression were indicated as the percentage decrease due to MTA co-incubation [(absolute value with MTA/ absolute value without MTA)*100]. CD16 expression levels (MFI) were analyzed on CD56dim NK cells within the two donor groups (D). Significance was calculated using a Mann-Whitney test for analyzing unpaired samples (n: 6). [file Image_5.TIFF]

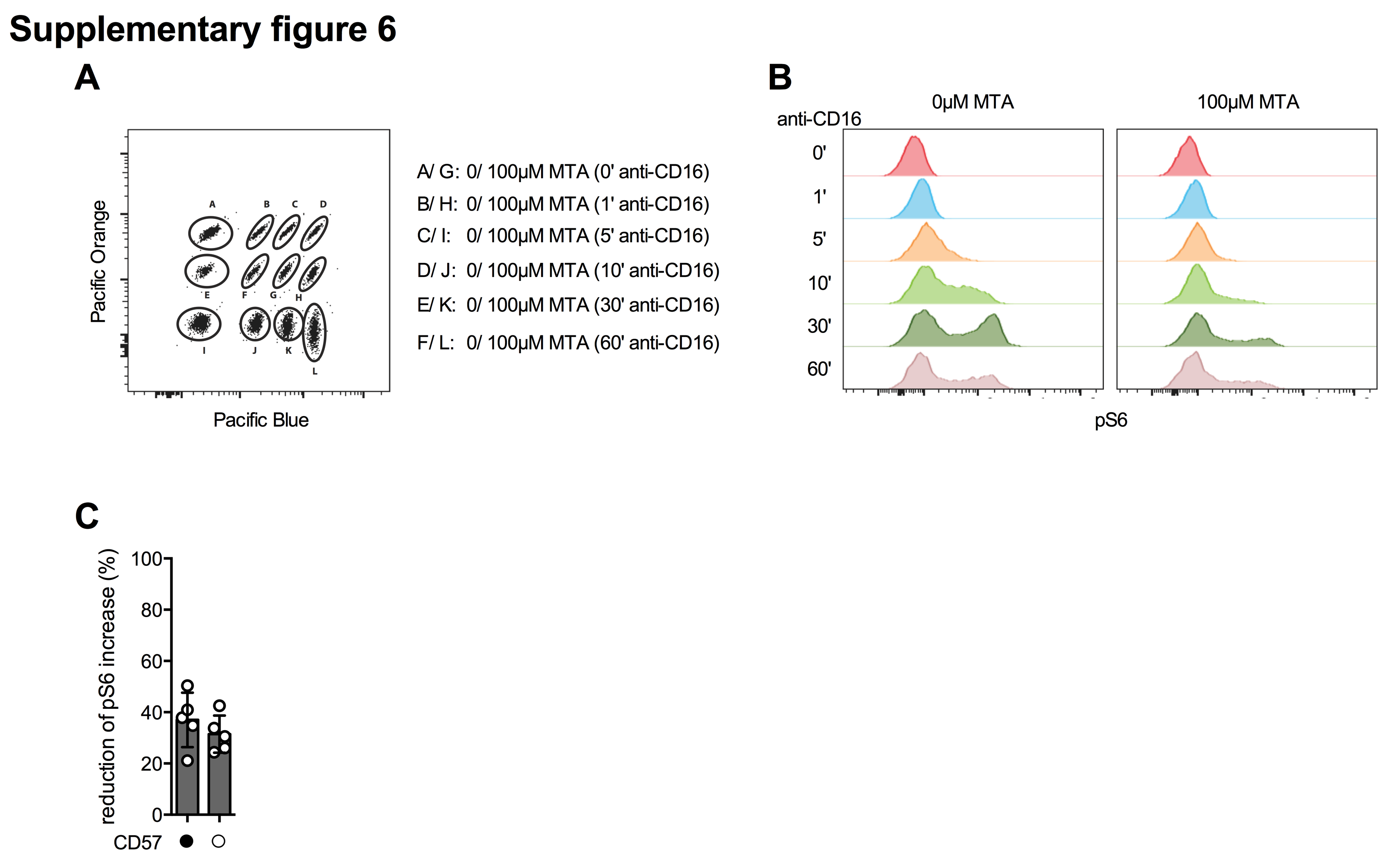

Supplement: Supplementary Figure 6 — MTA inhibits the NF-κB, PI3K/AKT/S6 and MAPK/ERK pathways down-stream of the CD16 receptor. Freshly isolated NK cells were pre-incubated with or without 100 μM MTA for 30′ at 37°C and then stimulated with anti-CD16 antibodies for 0, 1, 5, 10, 30, and 60′. Bulk (A,B) or CD57+/- NK cells (C) were labeled with a fluorescent bar cell code (A) and stained for the phospho-epitope S6 (B,C, n:5). [file Image_6.TIFF]
