# Supplementary material for: The Oncometabolite 5′-Deoxy-5′-Methylthioadenosine Blocks Multiple Signaling Pathways of NK Cell Activation
Source: Front Immunol. 2020 Oct 6;11:2128. doi: 10.3389/fimmu.2020.02128 (PMC7573074; doi:10.3389/fimmu.2020.02128)
Supplement: Supplementary Table 1 — Differential expression of adenosine receptors and methyltransferases in CD57+/− NK cells. [file Table_1.docx]

**Supplementary Table 1: Differential expression of adenosine receptors and methyltransferases in CD57^+/-^ NK cells**

| **Gene** | **Log2FoldChange** | **p-Value** | **FDR** |
| --- | --- | --- | --- |
| KMT2A.1 | 0,537000000000001 | 0,018595838 | 0,999557387008686 |
| NCAM1 | 0,406000000000002 | 0,026128356 | 0,999557387008686 |
| KMT5A.2 | 0,735666666666668 | 0,033620963 | 0,999557387008686 |
| PRMT2 | 0,310666666666668 | 0,04853923 | 0,999557387008686 |
| KMT5A | 0,599666667 | 0,04870359 | 0,999557387008686 |
| PRMT5 | 0,505 | 0,05556313 | 0,999557387008686 |
| PRMT6 | -0,290666667 | 0,120857432 | 0,999557387008686 |
| PRMT9 | 0,232 | 0,178893389 | 0,999557387008686 |
| CARM1 | -0,209 | 0,188607889 | 0,999557387008686 |
| KMT5A.1 | 0,162666667 | 0,25747889 | 0,999557387008686 |
| SETDB1 | -0,195666667 | 0,27605765 | 0,999557387008686 |
| PRMT3 | 0,325 | 0,276357581 | 0,999557387008686 |
| ADORA2A | -0,147666667 | 0,323561515 | 0,999557387008686 |
| EHMT2 | -0,119 | 0,435391608 | 0,999557387008686 |
| EHMT2.1 | -0,119 | 0,435391608 | 0,999557387008686 |
| PRMT8 | 0,1 | 0,444364493 | 0,999557387008686 |
| PRDM9 | 0,131 | 0,444654417 | 0,999557387008686 |
| ADORA1 | -0,098 | 0,486225167 | 0,999557387008686 |
| HNMT | -0,094 | 0,495485496 | 0,999557387008686 |
| PRMT1 | 0,092 | 0,514507105 | 0,999557387008686 |
| GNMT | -0,089666667 | 0,51884484 | 0,999557387008686 |
| DOT1L | -0,095 | 0,527595322 | 0,999557387008686 |
| DNMT1 | 0,102 | 0,563288704 | 0,999557387008686 |
| EZH2 | 0,084 | 0,58253794 | 0,999557387008686 |
| PRMT7 | -0,072666667 | 0,584503823 | 0,999557387008686 |
| NNMT | -0,084 | 0,584954826 | 0,999557387008686 |
| SETD2 | 0,076333333 | 0,592409954 | 0,999557387008686 |
| ADORA2B | 0,085666667 | 0,631555742 | 0,999557387008686 |
| KMT2A | 0,056666667 | 0,656970023 | 0,999557387008686 |
| GAMT | 0,058333333 | 0,722661842 | 0,999557387008686 |
| SUV39H2 | -0,072 | 0,730435265 | 0,999557387008686 |
| SMYD2 | -0,116666667 | 0,741417692 | 0,999557387008686 |
| PNMT | 0,043666667 | 0,770843393 | 0,999557387008686 |
| WHSC1 | -0,075666667 | 0,783245836 | 0,999557387008686 |
| EZH1 | -0,053333333 | 0,788140828 | 0,999557387008686 |
| EHMT1 | 0,028333333 | 0,868455307 | 0,999557387008686 |
| ADORA3 | -0,017 | 0,892653376 | 0,999557387008686 |
| TPMT | 0,016666667 | 0,920583367 | 0,999557387008686 |
| COMT | -0,003 | 0,983788804 | 0,999557387008686 |
